# EXPLORING THE LINK BETWEEN PLACE ATTACHMENT AND SOCIAL WELLBEING IN OLDER ADULTS

**DOI:** 10.1093/geroni/igae098.4186

**Published:** 2024-12-31

**Authors:** Mary Cox, Jennifer Beatty, Patrick Hill

**Affiliations:** Washington University in St. Louis, St. Louis, Missouri, United States; Washington University in St. Louis, St. Louis, Missouri, United States; Washington University in St. Louis, St. Louis, Missouri, United States

## Abstract

Many residents of St. Louis feel strong emotional bonds with the city, a phenomenon known as ‘place attachment.’ The relationship between place attachment and social well-being in older adults is a crucial area of study, particularly in an urban center like St. Louis, which has been the stage for important sociocultural events in Missouri. Research indicates that place attachment is positively correlated with social well-being and belonging. This connection is significant for older adults, as environments that promote social connection, autonomy, and security can support cognitive health and overall well-being in older adults. The current study tested our prediction that older adults with increased attachment to St. Louis will report higher levels of social well-being. Using survey data from 492 participants ages 65-85, we conducted correlational analyses to examine the relationship between intergroup anxiety and social well-being, exploring whether these associations differ based on levels of attachment to St. Louis. There were significant correlations between attachment to St. Louis (M=4.39, SD=1.31) and the well-being facets of social acceptance, social integration, social contribution, and social actualization (p< 0.001); however, social coherence was not significantly associated with place attachment. Moreover, place attachment was not associated with other wellbeing indicators, such as self-reported intergroup anxiety. While place attachment has beneficial social outcomes, it does not improve all wellbeing indicators. This suggests that a strong bond with St. Louis may not address all social challenges, highlighting the need for further exploration of how urban environments can better support older adults’ psychological and social needs.

